# The neurological disease ontology

**DOI:** 10.1186/2041-1480-4-42

**Published:** 2013-12-06

**Authors:** Mark Jensen, Alexander P Cox, Naveed Chaudhry, Marcus Ng, Donat Sule, William Duncan, Patrick Ray, Bianca Weinstock-Guttman, Barry Smith, Alan Ruttenberg, Kinga Szigeti, Alexander D Diehl

**Affiliations:** 1 Department of Philosophy, University at Buffalo, 135 Park Hall, Buffalo, NY 14260, USA; 2 Department of Neurology, University at Buffalo School of Medicine and Biomedical Sciences, 701 Ellicott Street, Buffalo, NY 14203, USA; 3 Department of Oral Diagnostic Sciences, University at Buffalo School of Dental Medicine, 355 Squire Hall, Buffalo, NY 14214, USA

## Abstract

**Background:**

We are developing the Neurological Disease Ontology (ND) to provide a framework to enable representation of aspects of neurological diseases that are relevant to their treatment and study. ND is a representational tool that addresses the need for unambiguous annotation, storage, and retrieval of data associated with the treatment and study of neurological diseases. ND is being developed in compliance with the Open Biomedical Ontology Foundry principles and builds upon the paradigm established by the Ontology for General Medical Science (OGMS) for the representation of entities in the domain of disease and medical practice. Initial applications of ND will include the annotation and analysis of large data sets and patient records for Alzheimer’s disease, multiple sclerosis, and stroke.

**Description:**

ND is implemented in OWL 2 and currently has more than 450 terms that refer to and describe various aspects of neurological diseases. ND directly imports the development version of OGMS, which uses BFO 2. Term development in ND has primarily extended the OGMS terms ‘disease’, ‘diagnosis’, ‘disease course’, and ‘disorder’. We have imported and utilize over 700 classes from related ontology efforts including the Foundational Model of Anatomy, Ontology for Biomedical Investigations, and Protein Ontology. ND terms are annotated with ontology metadata such as a label (term name), term editors, textual definition, definition source, curation status, and alternative terms (synonyms). Many terms have logical definitions in addition to these annotations. Current development has focused on the establishment of the upper-level structure of the ND hierarchy, as well as on the representation of Alzheimer’s disease, multiple sclerosis, and stroke. The ontology is available as a version-controlled file at http://code.google.com/p/neurological-disease-ontology along with a discussion list and an issue tracker.

**Conclusion:**

ND seeks to provide a formal foundation for the representation of clinical and research data pertaining to neurological diseases. ND will enable its users to connect data in a robust way with related data that is annotated using other terminologies and ontologies in the biomedical domain.

## Background

Neurology is concerned with diseases related to the functioning of the nervous system. These diseases may present acutely or chronically, have transient or progressive courses, and affect a variety of anatomical regions and cell types. They are realized through diverse mechanisms, including cell-autonomous disorders, unregulated protein aggregation, autoimmune conditions, or vascular pathology [1,2]. There are a variety of ways to classify neurological diseases, such as by symptomology or pathology. Several classificatory systems and terminologies are currently available, such as NIFSTD, ICD-10, SNOMED CT, MeSH, and the Disease Ontology [3-7]. Although some of these classificatory systems and terminologies are widely used for purposes such as billing and medical messaging, they do not satisfy current best practices in ontology development and do not provide the level of detail needed for precise annotation of and reasoning over data.

Definitions for terms in medical terminologies are often ambiguous or vague, and often the meanings of such terms are defined for use only in one particular domain. Clinical or research data is thus rarely encoded in a way that will allow the linking of various types of data together in a coherent fashion that will also support computation. Yet, computer-aided reasoning has become increasingly important to medical research due, in part, to the vast amount of data being generated [8]. This means that it is more important than ever for data to be annotated in a clear and unambiguous manner in order to facilitate integration across diverse sources and thereby maximize the benefit of scientific investigation. The Open Biomedical Ontology (OBO) Foundry promotes the development of consistent formal ontologies based on a common upper-level reference ontology to address this need [9]. The success of the Gene Ontology has shown how a controlled and properly curated ontology can benefit and extend research in medicine [10].

The Neurological Disease Ontology (ND) is being developed as an extension of the Ontology for General Medical Science (OGMS). OGMS represents entities in the domain of medicine and disease and addresses the need to integrate biomedical data [11,12]. The OGMS framework consists of approximately 100 terms that describe fundamental aspects of medicine, such as ‘disorder’ , ‘diagnosis’ , ‘disease course’ , ‘clinical encounter’ and ‘syndrome’. OGMS utilizes a template for generating unambiguous textual and formal logical definitions for terms in an effort to promote the integration of scientific data. ND seeks to produce definitions in a similar manner within the domain of neurological disease.

We are developing the Neurological Disease Ontology to provide a framework for the representation of key aspects of neurological disease. ND is compliant with OBO Foundry principles and builds upon the paradigm established by OGMS. ND is an ongoing collaborative project that aims to establish a formal structure to enable precise representation of a variety of neurological diseases and disorders. Our ultimate goal is to accurately represent for each disease its molecular, genetic and environmental origins, the processes involved in its etiology and course of progression, as well as its clinical presentation and phenotypes, including associated signs, symptoms, syndromes, diagnostic criteria, treatment, and testing methods. ND has three initial areas of focus:

1. Alzheimer’s disease and diseases resulting in dementia

2. multiple sclerosis and demyelinating diseases

3. stroke and cerebrovascular events

## Construction and content

### Development of ND

ND is being developed using both a top-down and bottom-up approach to term creation. We use the top-down approach to create high-level classes in ND by analyzing the types of neurological diseases presented in clinical literature and determining how to classify them within the ontology. This approach involves determining what additional core entities should be part of the ND framework in order to allow for a more complete representation of the domain, including relationships between upper-level classes. NIF_Dysfunction was used as starting point for developing the class hierarchy for ‘neurological disease’ in ND [13]. The bottom-up approach involves reviewing primary research articles, review articles, texts and other sources to inform the development of ND. Domain experts and clinical collaborators provide constructive feedback and guide decision making on controversial material. We examine samples of available data sources, such as forms for recording clinical history, functional assessments, and diagnostic charts, to let the data inform term creation and refinement. This approach has provided the majority of classes and definitions in ND.

**Figure 1:**
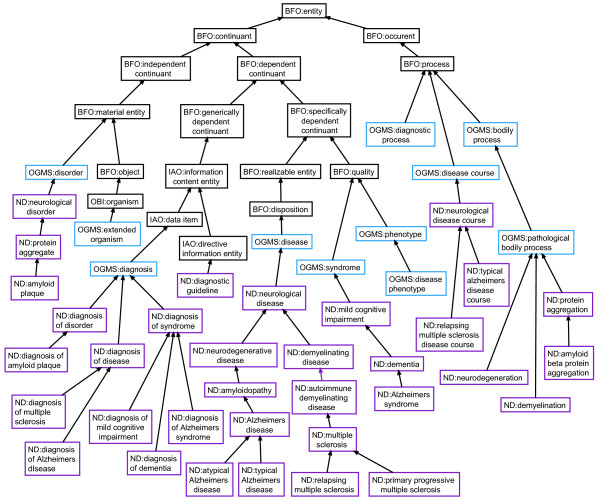
**Graphical overview of high level terms in ND.** A subset of classes in ND, showing the *is_a* relationships between BFO (in black), OGMS (in blue) and ND (in purple).

**Figure 2:**
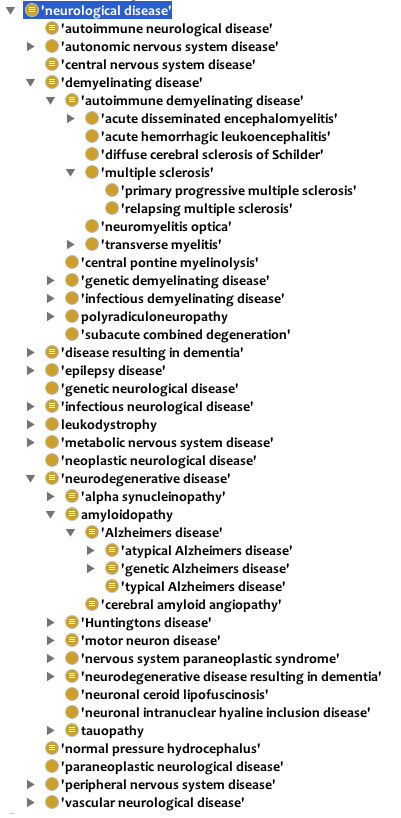
**Classification of neurological diseases.** Screen shot from Protégé showing neurological disease hierarchy.

**Figure 3:**
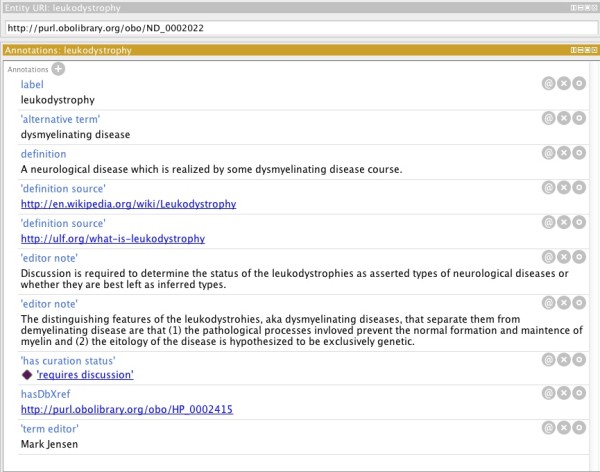
**Annotations of ND terms.** Screen shot from Protégé showing annotations for the ND class ‘leukodystrophy’.

The ontology currently contains approximately 450 ND classes. Primary work has been done under the OGMS classes ‘disease’ , ‘diagnosis’ , ‘disease course’ , and ‘disorder’. Figure 1 shows a subset of classes illustrating a portion of the asserted *is_a* hierarchy between ND, OGMS, and the Basic Formal Ontology (BFO) [14]. In particular, we have built a large hierarchy of subclasses of ‘neurological disease’ as illustrated in Figure 2. The 182 subclasses of ‘neurological disease’ are further subdivided into additional classifications such as ‘neurodegenerative disease’ , ‘demyelinating disease’ , and ‘autoimmune neurological disease’. We assert a single inheritance hierarchy of disease and capture the complexity of many neurological diseases through the use of logical definitions that capture additional disease characteristics as ontological differentia (see below). We have also imported over 700 classes from external ontologies, such as the Protein Ontology (PR), Foundational Model of Anatomy (FMA), and Ontology for Biomedical Investigations (OBI) [15-17]. ND terms are annotated with ontology metadata such as a label (term name), term editors, definition, definition source, curation status, and alternative terms (synonyms). Currently there are over 300 external references to other ontologies and vocabularies. Figure 3 shows a sample of the annotations for a term in ND.

We attempt to provide both textual and logical definitions for every class. A logical definition provides a set of formal axioms specifying the connections between related ontology classes, enabling formal reasoning using the ontology. These are essential in order to provide meaningful results when performing analysis on data annotated using ND. Currently, approximately 50% of the classes in ND have logical definitions. Textual definitions attempt to capture the logical definitions using natural language and provide additional clarification when needed. Editor notes are used to offer alternative definitions, indicate gaps in current knowledge, or provide further elucidation beyond that of the textual definition. We adhere to the principle of ontological realism in developing ND [18]. Wherever possible, terms in ND refer to universal types. Some ND terms, such as ‘vascular disease resulting in dementia’, have been created for organizational purposes and are identified as such in their annotations.

ND is being developed using Protégé 4.x and implemented in OWL 2 [19,20]. The development versions of OGMS ( http://purl.obolibrary.org/obo/ogms/dev/ogms.owl) and BFO 2 ( http://purl.obolibrary.org/obo/bfo.owl) provide the upper-level classes and relations for ND [21]. ND imports classes from other ontologies following the MIREOT standard using OntoFox [22]. All relationships discussed are type-type relationships of the form “X RELATION *some* Y”, such that for every instance x of the type X, x necessarily must stand in relation to some instance y of the type Y.

### ND as an extension of OGMS

In addition to classifying, naming, and defining diseases, development of ND requires representation of entities related to diseases, such as the disease course and symptomology. ND is being developed as an extension of OGMS, which provides essential high-level terms including ‘disease’ , ‘disorder’ , and ‘disease course’. OGMS defines ‘disease’ as a disposition of an organism to undergo pathological processes that exists because of one or more disorders of that organism (OGMS_0000031). A disposition is a realizable entity that inheres in a material entity and will manifest particular behaviors given certain operative conditions. Thus, diseases are propensities for certain pathological processes to occur as a result of a material disorder in an organism. Figure 2 shows the class hierarchy for ND ‘neurological disease’ , which is a subclass of OGMS ‘disease’.

In general, we create textual definitions for terms based on the Aristotelian format according to which each class is defined as a member of its parent class plus a differentiating criterion. The following definitions of core ND terms exemplify this approach: ‘neurological disease’ is “A disease that has material basis in a neurological disorder and, when realized, the resultant pathological processes affect functioning of the nervous system” (ND_0000001); ‘demyelinating disease’ is “A neurological disease that is characterized by loss or dysfunction of myelin in the central or peripheral nervous system” (ND_0000024); ‘neurodegenerative disease’ is “A neurological disease that is characterized by atrophy or death of neurons or related structures progressively affecting the functioning of the nervous system” (ND_0000113); ‘neurological disorder’ is “A disorder that is the material basis of a neurological disease” (ND_0005501).

OGMS represents the manifestation of a disease as a complex set of interrelated entities using multiple ontological classes. One area of development in ND has been the extension of OGMS ‘disease course’ , which is defined as “the totality of all processes through which a given disease instance is realized” (OGMS_0000063). In developing ND, parts of certain disease course types are specified and defined. For example, an instance of ‘amyloidopathy disease course’ is the realization of an instance of ‘amyloidopathy disease’. Thus, the definition of ND ‘amyloidopathy disease course’ (ND_0006031) includes the pathological process ‘amyloid beta protein aggregation’. The formal definition for ‘amyloidopathy disease course’ contains this necessary condition:

‘amyloidopathy disease course’ has_occurrent_part *some* ‘amyloid beta protein aggregation’

To connect this class to other classes in the ontology additional conditions are asserted:

‘amyloidopathy disease course’ realizes *some* ‘amyloidopathy’

‘amyloidopathy disease course’ has_participant_at_all_times *some* (‘extended organism’ and bearer_of_at_all_times’ *some* ‘amyloidopathy’)

**Figure 4:**
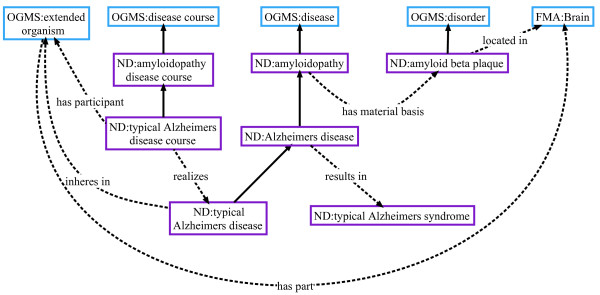
**Ontological representation of amyloidpathy and Alzheimer’s disease.** Solid black arrows indicate *is_a* relationship.

For example, any process that instantiates ‘Alzheimers disease course’, a subtype of ‘amyloidopathy disease course’, is automatically related to an instance of ‘Alzheimers disease’, the organism participating in this process, and the material basis. Figure 4 illustrates and elaborates on these connections.

ND distinguishes syndromes from the diseases that produce them. OGMS defines ‘syndrome’ as “A pattern of signs and symptoms that typically co-occur” (OGMS_0000086). Syndromes, as we interpret them according to a commonly held view, share observable clinical presentations – features that are often recognizable as being related and predictive. Yet syndromes do not always have common underlying causes and more than one disease type may result in instances of the same syndrome. For example, we classify dementia as a syndrome and not a disease. ND ‘dementia’ is a subtype of ND ‘mild cognitive impairment’ and is defined as “The co-occurrence of cognitive deficits in at least two domains [memory, visual spatial, attention, orientation, executive function, functional] resulting in a decline of previous functioning” (ND_0003000). Dementia can result from numerous diseases including Alzheimer’s, Parkinson’s, and Lewy body disease.

A syndrome is a BFO ‘quality’ and as such must be borne by a material entity, e.g., an organism. The organism is also the bearer of the disease that gives rise to the pathological processes that produce signs and symptoms, some of which constitute the syndrome. A complex set of axioms is required to relate a disease to a syndrome that results from the disease. ND introduces and defines ‘results in’ as a shortcut relation [23,24] to capture the disease-to-syndrome link. ND ‘results in’ has domain ‘disease’ and range ‘syndrome’ and is defined as:

‘disease’ D results_in ‘syndrome’ S = _def_ D is_realized_by ‘disease course’ C *and* C has_specific_dependent_at_some_time S *and* C has_participant ‘organism’ O *and* O is_bearer_of (D *and* S)

### Support for inferential reasoning in ND

The use of consistent logical definitions allows additional hierarchies and relationships between terms to be inferred via automated reasoning. In developing ND, we chose to construct the asserted disease hierarchy according to the primary mechanism for each disease. This reflects our understanding of diseases and their various axes of differentiation based on current methods in medicine, such as advanced imaging techniques and genetic research. Alternatively, the asserted hierarchy could have been developed based on criteria such as the source of the disease (e.g. genetic vs. acquired diseases), the affected physiological region(s) (e.g. according to brain regions or central vs. peripheral parts of the nervous system), or the resulting symptomology. Many diseases also have idiopathic or secondary mechanisms, such as infections or environmental exposures that may precede certain cases of multiple sclerosis [25]. Since we do not allow for multiple inheritance in the construction of the ontology, we encode secondary differentia via carefully defined axioms, so that with the assistance of logical reasoners, we may query for information about, for example, diseases that have a material basis in the spinal cord or diseases that have infections associated with their etiology.

ND ‘Alzheimers disease’ is “An amyloidopathy where cognitive deficits [memory, visual spatial, attention, orientation, executive function, functional] occur that represent a decline from previous levels of functioning resulting in dementia caused by neurodegeneration as a result of deposition of amyloid plaques and neurofibrillary tangles in the medial temporal lobes” (ND_0000152). ‘Alzheimers disease’ is thus considered to be a type of ‘amyloidopathy’. This reflects the most current theories about the underlying disorder for Alzheimer’s disease (AD). AD is a member of a family of diseases that result from the unregulated accumulation of amyloid protein. However, a significant part of the material basis of AD is also neurofibrillary tangles; also called tau tangles [26,27]. It shares this in common with other tauopathies. To obtain the inferred relationship of ‘Alzheimers disease’ also being a kind of ‘tauopathy’, the logical specification for ‘Alzheimers disease’ has the necessary condition:

‘Alzheimers disease’ (has_material_basis_at_all_times *some* ‘amyloid beta plaque’) *and* (has_material_basis_at_all_times *some* ‘neurofibrillary tangle’)

**Figure 5:**
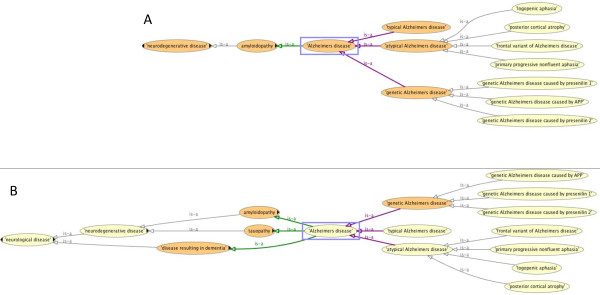
**Asserted vs. inferred hierarchy for Alzheimer’s disease. (A)** asserted hierarchy for ‘Alzheimers disease’ and **(B)** inferred hierarchy for ‘Alzheimers disease’ where the *is_a* links to ‘tauopathy’ and ‘disease resulting in dementia’ are inferred.

All tauopathies have material basis in some neurofibrillary tangles and when a reasoner is run on the ontology, ND ‘Alzheimers disease’, along with any disease class that has an asserted material basis of neurofibrillary tangles, will be inferred to be a ‘tauopathy’ in addition to its asserted parent. Figure 5 shows the asserted vs. inferred hierarchy for ND ‘Alzheimers disease’. Other examples of alternate ways of classifying diseases through inference are ND ‘central nervous system disease’ or ND ‘disease resulting in dementia’. No disease types are asserted to be children of these classes; rather, these classes are used for reasoning purposes. They are organizational terms that provide another way of classifying diseases and related entities. As discussed below, adding terms such as these to the ontology will aid in developing queries and allow novel questions to be asked using the ontology for our use cases.

## Utility and discussion

ND is developed as a collaborative project, both for clinical applicability and for coordination and unification of a variety of biomedical ontologies for research purposes. Many disease ontologies exist that offer unique representations of a single disease or family of diseases. This has produced overlapping representations. For example, some neurological diseases are also infectious diseases. By adhering to the OGMS framework, ND is consistent with and is developed collaboratively with other ontologies using OGMS. These include the Infectious Disease Ontology (IDO), Mental Functioning (MF) and Mental Disease (MD) Ontologies, Adverse Event Reporting Ontology (AERO), and Ontology of Medically Related Social Entities (OMRSE) [28-31]. For example, we have proposed that our relation ‘results in’ – used to connect a disease to a syndrome – be added to OGMS so that it is readily available for use by other OGMS-compliant disease ontology efforts.

In addition to consistently classifying neurological diseases according to their primary disease mechanism and then identifying the relevant associated entities following the OGMS model, we have sought to define the relationships that hold between these entities using formal logical definitions for each class in ND. This will facilitate integration of data resources and provide accurate annotation and advanced querying capabilities across data from a variety of domains within the general field of neurology. This includes, for example, relating genomic data to patient reported outcomes and cognitive testing in the treatment and study of multiple sclerosis or Alzheimer’s disease, as well as providing the proper formal framework to allow for logical reasoning. Reasoning with datasets through the use of logical relations is a capability that other ontologies in the domain of neurological disease have not fully explored. Other efforts exist that have used ontologies to annotate and query over datasets, such as the Sleep Domain Ontology and Epilepsy and Seizure Ontology [32,33]. However, these ontologies focus on entities that are specific to their respective domains rather than on neurological diseases in general. Although these ontologies have proven valuable for their specific use cases, they differ from our goal to develop a domain-encompassing ontology for neurological diseases that utilizes relations between the disease classes and related classes to maximize the potential reasoning power. ND is being built to provide both an accurate description of neurological diseases as well as to serve clinical and research purposes through annotation and reasoning. A key developmental goal is to create a more thorough representation of neurological diseases than can be expressed using only an *is_a* hierarchy. This is one way in which we build upon prior efforts in this area, such as NIF_Dysfunction [3].

A key decision during the initial phase of development regarded the extent to which the NIF_Dysfucntion hierarchy should be maintained or altered in ND. While we believe it is important to utilize existing work as much as possible, we chose to not directly import NIF_Dysfunction for several reasons. Generally we found the NIF_Dysfunction hierarchy in need of further curation. More specifically, there were several inconsistencies in the hierarchy that made direct use problematic – especially given our commitment to develop a comprehensive OGMS-based picture of neurological disease. Distinctions between upper-level classes in NIF_Dysfunction are made based on disease mechanism, while lower-level distinctions are made based on symptomology [34]. Ambiguous uses of the terms ‘disease’ , ‘syndrome’ , and ‘disorder’ exist throughout NIF_Dysfunction. For example, the term ‘Cerebrovascular Disorder’ is a subclass of ‘Nervous system disease’, but the children of ‘Cerebrovascular Disorder’, such as ‘Brain Ischemia’ and ‘Cerebral Hemorrhage’ , are not diseases. There is also a term for ‘Nervous System Trauma’ under ‘disease’. A clear distinction needs to be made between physical abnormalities and injuries versus diseases. Also, many disease types in NIF_Dysfunction are better understood as symptoms or signs, like ‘paralysis’ , ‘paresis’ , and all of the subtypes under ‘dyskinesia’. We have held preliminary discussion with NIF_Dysfunction developers to determine whether they would alter their hierarchy to match ours or develop bridging axioms to connect the two ontologies. The goal is not to replace, but rather to connect to and integrate various disease ontologies such as NIF_Dysfunction and the Disease Ontology (DO), as well as related ontologies such as the Phenotypic Quality Ontology (PATO) [35,36]. These ontologies all use BFO for their upper level terms and adhere to OBO Foundry principles, yet offer differing classifications of diseases and related entities.

**Figure 6:**
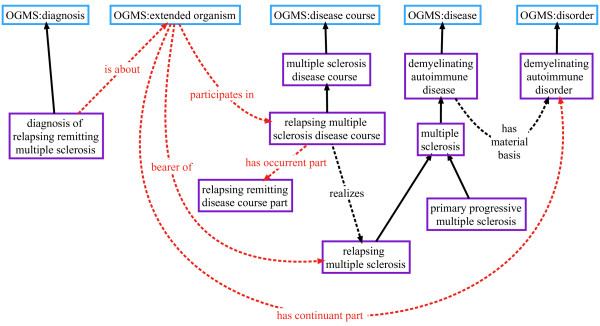
**Ontological representation of diagnosis of relapsing remitting multiple sclerosis.** With the exception of the solid black lines indicating *is_a* relationships, all other relationships shown are between instances of classes in the ontology. Red dashed lines indicate axioms that are asserted for the diagnosis class only, not universal truths about any entity other than the diagnosis.

In addition to providing the framework for clear annotation and reasoning over data, the approach for representing disease we adapt from OGMS resolves a great deal of ambiguity. For example, terms in different vocabularies often describe unique disease types for entities that are better understood as a stage or part of a disease course. A common error made by multiple sclerosis (MS) terminologies is to assert the existence of unique disease types for the clinical variants of MS: relapsing remitting multiple sclerosis (RRMS), secondary progressive multiple sclerosis (SPMS), and primary progressive multiple sclerosis (PPMS). Research into this domain strongly suggests that these variants are distinct phases in the realization of MS and are not themselves unique types of disease [37,38]. These variants are distinguished by their unique patterns of episodes of worsening neurological functioning both in terms of duration and intensity, which can be charted over time. Disability due to MS is measured according to the Kurtze Expanded Disability Status Scale (EDSS), which produces an identifiable and unique pattern of neurological disability. For example, RRMS is a part of the relapsing multiple sclerosis disease course that is specified by a particular relapsing-remitting pattern of neurological disability, which can be specified in a formal manner. When a clinical variant of MS is referred to in a dataset, say, as an instance of a diagnosis of RRMS, we annotate it in ND by generating an instance of ‘diagnosis of relapsing remitting multiple sclerosis’ , which is about an organism that bears the disease relapsing multiple sclerosis and participates in a relapsing multiple sclerosis disease course. Figure 6 illustrates this by showing the relationships that exist between instances of classes in the ontology whenever an instance of ‘diagnosis of relapsing remitting multiple sclerosis’ is created.

### Ongoing applications of ND

ND is being built with an eye toward the annotation of data in the pursuit and support of novel research. In support of one use case, we recently received a grant from the National Multiple Sclerosis Society to investigate the relationship between patient-reported outcomes and clinical findings for MS as reported in the New York State Multiple Sclerosis Consortium patient registry, which has been collecting MS patient data for nearly two decades [39]. We will be adding additional terms to ND to support this application and then use terms from ND to annotate de-identified patient records. Using our corpus of annotated records, we will be able to query for specific subsets of patient records based on criteria such as the type of MS disease course or type of treatment. We will then look for unexpected co-annotated terms, particularly with respect to treatments and clinical outcomes. For instance, we will look for unexpected outcomes in certain subsets of patients given particular treatments or syndromes associated with disease courses that share certain pathological processes.

We are also collaborating with our clinical partners at the University at Buffalo to develop terms in ND in support of patient registries for Alzheimer’s disease and stroke victims. This work has led to the development of a corollary project to ND, the Neuropsychological Testing Ontology (NPT) [40]. NPT is an extension of the Ontology for Biomedical Investigations (OBI) [17] that represents the kinds of neuropsychological tests used in assessing cognitive functioning in patients with a variety of diseases, injuries and conditions. One area of future development in ND is within the diagnosis branch of the ontology. Since neuropsychological tests are used in diagnostic processes in neurology, there is need to link the two ontologies, ND and NPT, in order to connect clinical findings, such as testing results, to diagnostic criteria for neurological diseases.

## Conclusions

The Neurological Disease Ontology is being generated and curated according to best practices in ontology development. We are adhering to fundamental principles of reuse and collaboration to maximize data integration. We are providing a set of reference terms applicable to any neurological disease as well as developing specific extensions for AD and MS. These extensions provide templates for others interested in building additional extensions of ND. ND is an important first step toward achieving the goal of linking the material basis of neurological diseases to their symptomatology as manifested in particular patients using a consistent, controlled, and unambiguous reference ontology. ND provides an excellent foundation to represent clinical and research data, and formally connects it with related data from other domain terminologies and ontologies across the spectrum of biomedical informatics.

ND is under active development. As we continue to work on the ontology, we expect to make many additions as well as needed revisions in order to better represent the domain. Further work in ND will involve continued development of the representation of material bases, treatments, phenotypes, and associated syndromes of neurological diseases as well as exploration of the links between diagnoses, diagnostic guidelines, and clinical findings. We welcome collaborators interested in developing disease-specific extensions or in using ND for annotation and reasoning purposes.

## Availability and requirements

ND is freely available under the New BSD License (Code) and the Creative Commons 3.0 BY License (Content) at the following URL:

http://neurological-disease-ontology.googlecode.com/svn/trunk/src/ontology/ND.owl

## Competing interests

The authors declare that they have no competing interests.

## Authors’ contributions

All authors contributed to research and development of the ontology. APC and MJ are the primary curators of the ontology. ADD manages the project. NC and MN did preliminary research on stroke and cerebrovascular disease and DS incorporated this information into the ontology. WD and AR provided helpful technical support and creative feedback. KS and BWG are clinical collaborators. MJ drafted the manuscript with contributions from ADD, APC, PR and BS. All authors read and approved the manuscript.
